# Study of the relationship between transposable elements and microRNAs in the development of glioblastoma for its targeted therapy

**DOI:** 10.37349/etat.2026.1002408

**Published:** 2026-10-20

**Authors:** Rustam Mustafin

**Affiliations:** Fondazione Policlinico Universitario Agostino Gemelli IRCCS, Italy; Department of Medical Genetics and Fundamental Medicine, Bashkir State Medical University, 450008 Ufa, Russia

**Keywords:** circular RNAs, glioblastoma, lncRNAs, microRNAs, targeted therapy, transposable elements

## Abstract

Glioblastoma (GB) differs from other malignancies in its pronounced aggressiveness, drug resistance (including with modern chemotherapy and anti-PD-1 immunotherapy), high mortality, and the involvement of transposable elements (TEs) in its development. This is reflected in the high frequency of detection of TE-driven chromothripsis in GB samples (39%). Chromothripsis affects chromosomes 7, 9, and 12, with amplification of the *EGFR* and *MDM2/CDK4* oncogenes. GB is characterized by the expression of multiple transcripts and peptides derived from specific TEs and stimulating an antitumor immune response. At the same time, LINE1 and a number of long terminal repeat-containing retroelements have been described as having a tumor-promoting effect on GB. Therefore, a differentiated approach to treating GB is promising, with inhibition of TEs involved in carcinogenesis and simultaneous activation of the safest and most immunogenic TEs (viral mimicry). For this approach, it has been proposed to use microRNAs derived from TEs and represented in the MDTE database. A review of the scientific literature identified 43 such microRNAs with tumor suppressor properties. However, additional experimental and clinical studies are needed for their possible use before these molecules can be considered as therapeutic targets. Twenty oncogenic microRNAs derived from TEs were also identified. Interactions of these microRNAs with long noncoding RNAs and circular RNAs in GB pathogenesis are also described.

## Introduction

Glioblastoma (GB) is the most common primary malignant neoplasm (MN) of the brain in adults and is characterized by marked resistance to existing treatments, including chemotherapy with temozolomide (TMZ) [1]. The proportion of GB among all MNs of the brain is 47.1%, the age of manifestation of GB is 45–70 years, and the prevalence is on average 3.2:100,000 and is associated with aging (in people over 85 years old 85.39:100,000). GB is classified into four subtypes based on their transcriptional profiles: proneuronal, neuronal, mesenchymal, and classical. Treatment for GB involves surgical excision with postoperative adjuvant radiation and chemotherapy (the cytotoxic alkylating agent TMZ). However, despite modern treatment methods, survival within 1 year after diagnosis is 36%, and for more than 3 years after diagnosis, survival is only 3–5%. The median survival for GB is 15 months [2]. At least 90% of patients with hypertension experience a relapse within 2 years, regardless of the initial response to previous therapy [3].

GB is a multifactorial disease, with both environmental factors and genetic predisposition playing a role in its development. Results from genome-wide association studies (GWAS) indicate an association between various polymorphic variants, including 18 single nucleotide polymorphisms (SNPs) located near genes involved in carcinogenesis, such as *TP53, TERT, EGFR, CDKN2B-AS1, RTEL1, CCDC26, PHLDB1,* and *ETFA* [4]. Most of the polymorphisms associated with GB are located in the intergenic, intronic and promoter regions of genes, which is typical for multifactorial diseases [5]. These loci contain the majority of transposable elements (TEs), which occupy almost half of all DNA sequences in the human genome [6] and are drivers of epigenetic regulation, which include DNA methylation, histone modifications, and RNA interference under the influence of noncoding RNAs (ncRNAs) [7]. Accordingly, GB-associated polymorphisms may cause deregulation of TEs, which is reflected in changes in epigenetic factors.

Indeed, the GWAS analysis of DNA methylation revealed 1,548 CpG sites (1,307 genes) differentially methylated in GB compared to normal brain tissue [8]. A reflection of the disturbances in the regulation of TEs during the development of GB is also a change in the expression of many microRNAs [9, 10], since TEs are important sources of their genes. An microRNAs derived from TEs (MDTE) database was created that reflects 405 such human microRNAs [11]. Additional factors of TEs activation that predispose to the development of GB are aging [2] and stress factors, since TEs are highly sensitive sensors of the genome to environmental influences [12]. In addition, there is evidence directly implicating hyperactivated TEs in the development of GB. Based on the above, a hypothesis has been proposed that TEs, activated under the influence of specific polymorphisms (associated with GB), aging, and stress, participate in the development of GB through various pathways. The primary effect of TEs is due to their carcinogenesis stimulating by promoting genomic instability, inactivating tumor suppressor genes, and activating oncogenes. Additional effects may be associated with stimulation of the antitumor immune response by transcripts and proteins of certain immunogenic TEs activated during the development of GB. Since TEs are important sources of microRNAs and other ncRNAs, altered TE activity inevitably causes global epigenetic disruptions and altered microRNA expression. At the same time, complete complementarity of microRNAs to TE sequences may provide the basis for designing specific targeted therapies aimed at influencing TEs involved in the development of GB. Therefore, the use of specific microRNAs is promising for balancing the immune response stimulated by TE proteins and transcripts (they require derepression) and the oncogenic activity of TEs (they require inhibition of expression).

### Search strategy

To write this review, a search strategy was used in the PubMed, Scopus, and WoS databases, specifying the phrases “glioblastoma transposable elements,” “glioblastoma microRNA,” “glioblastoma associated genes,” “glioblastoma viral mimicry,” “glioblastoma epigenetic therapy,” and “glioblastoma targeted therapy.” In addition, the scientific literature was analyzed to identify the involvement of all microRNAs represented in the MDTE database in glioblastoma development (for each TE-derived microRNA, the microRNA name was entered in combination with the word “glioblastoma”). Literature from 2001 to 2026 was analyzed. Sources describing the mechanisms of TE involvement in glioblastoma development, as well as TE-derived microRNAs, were selected. The objectives of this study are to determine the direct effects of TEs on the development of glioblastoma, the mechanisms of these effects, the potential for glioblastoma therapy by influencing TEs, and to determine the relationships of TEs with various ncRNAs. The novelty of this study is the description of the relationship of TEs with epigenetic factors in the development of glioblastoma, in particular, with oncogenic and oncosuppressive microRNAs.

## Direct effects of transposable elements on glioblastoma development

TEs are classified into class I—retroelements (REs), which use RNA as an intermediate for reverse transcription of complementary DNA (cDNA) inserted into the genome. Class II—DNA transposons, which use the “cut and paste” method of inserting their own genes into new DNA loci [13]. REs are the most common in the human genome. They comprise at least 41% of all nuclear DNA sequences and are divided into those containing long terminal repeats (LTRs) and those without LTRs. The latter include the most common autonomous REs, long interspersed nuclear elements (LINEs), which make up approximately 21% of the genome, and non-autonomous short interspersed nuclear elements (SINEs, primarily composed of Alus, which occupy 10.5% of the human genome). Most LTR REs are classified as endogenous retroviruses (ERVs), which occupy approximately 9% of the genome [6].

One of the reasons for the activation of TEs during GB progression may be stressful changes, such as hypoxia, since TEs are highly sensitive structures of the human genome to stress effects [12]. A significant increase in the number of SINE and LINE transcripts encoding reverse transcriptase was determined in GB cells of the U87MG line under the influence of hypoxia (cultivation in an atmosphere of 0.1% oxygen for 6 weeks). These reactions were due to significant hypomethylation of SINE loci and led to increased genomic instability under hypoxic conditions [14]. Comparative analysis of tumor samples taken from patients revealed lower LINE1 methylation in GB compared with low-grade gliomas and normal brain tissue. LINE1 methylation correlated with *O*^6^-methylguanine-DNA methyltransferase (MGDM) promoter methylation, being a good tumor prognostic factor [15]. In another study, higher levels of global LINE1 methylation were also correlated with longer survival time in patients with GB [16]. The obtained data indicate a stimulating effect of LINE1 on the development of GB (since methylation inhibits expression). Accordingly, artificial suppression of LINE1 may become one approach for targeted therapy of GB.

Sequencing of GB tissues from patients identified an endonuclease-dependent LINE1 insertion and LINE1-associated rearrangements, as well as Alu-Alu recombinations near LINE1. LINE1 insertions were identified in introns of the *EGFR* and *MeCP* (epigenetic regulator) genes [17]. In a study of GB samples from patients, stable LINE1 methylation status resulted in increased overall survival compared to unstable status. This suggests that abnormal LINE1 activation plays a role in the development of GB under the influence of epigenetic changes [18]. A computational method, REdiscoverTE, was developed to assess genome-wide TE expression based on RNA sequencing data. Using The Cancer Genome Atlas database, increased expression of over 400 TE subfamilies was identified in GB, 262 of which resulted from direct DNA methylation loss. Activation was most frequently detected for evolutionarily young TEs located in intergenic loci and associated with the antiviral response and reactions to DNA damage [19].

A study of GB tissues taken from patients revealed 48 LTR-containing TEs (34 of which belong to the ERV1 superfamily), the expression of which significantly differed from that of normal brain. Of these, 43 TEs showed increased expression [20]. The role of human ERVs (HERVs) in maintaining the phenotype of GB stem cells has also been determined [21]. Overexpression of multiple HERVs, including the HML-6 transcript localized to 19q13.43b, was detected in GB cell lines. The endogenous retrovirus HML-6 contains an intact open reading frame (ORF) encoding the ERVK3-1 envelope protein. High levels of ERVK3-1 in patients with GB were associated with a poor prognosis (regardless of *IDH* gene mutations). Therefore, the ERVK3-1 protein has been proposed as a potential therapeutic target for the treatment of GB [22].

Pathological activation of HML-2 (belongs to the HERV-K subgroup) was detected in GB tissues and in the cerebrospinal fluid of GB patients and was associated with a cancer stem cell phenotype and poor prognosis. Single-cell RNA sequencing identified GB cell populations with elevated HML-2 transcript levels in neuronal progenitor cells, which stimulated cellular plasticity [23]. Metagenomic analysis confirmed the expression of HERV-K113 (HML-2) in GB tissues, which was not detected in normal brain samples [24]. An analysis based on The Cancer Genome Atlas identified a locus-specific HERV transcriptome for GB, identifying 211 HERVs with significant dysregulation in GB compared to normal brain tissue. An algorithm was developed to uniquely evaluate HERVs specific to GB, glioma, and brain tissue. In this study, lower expression of HERVs correlated with worse survival in GB patients [25].

## Mechanisms of transposable elements influence on glioblastoma development

The study of the possibility of using TEs as targets for targeted therapy is due to their key role in carcinogenesis and the progression of genomic instability with its global rearrangements in the form of chromothripsis [26], which is observed in 39% of GB cases, while for other MNs its share is on average 9% [27, 28]. Pathologically activated TEs in carcinogenesis move to new loci, activating oncogenes and inactivating tumor suppressor genes [29], which is characteristic of chromothripsis in GB. Typically, GB samples are found to have homozygous deletion of *CDKN2A* and *PTEN* gene mutations, *TERT* promoter mutation, and *EGFR* oncogene amplification with an increase in the number of copies of chromosome 7 [30]. In 20% of patients with GB, additional amplifications of *MDM2, MDM4* and *CDK4* oncogenes are detected [28]. In GB tissues with chromothripsis, damage to chromosomes 7, 9, 12 with amplification of *MDM2/CDK4, EGFR* oncogenes and homozygous deletion of *CDKN2A* gene has been described [31]. GB is also characterized by chromosomal rearrangements, such as deletions of 10q23 and 10q25-26, which are used as markers for the development of GB and its aggressiveness [32].

Epigenetically activated TEs during the development of GB can influence oncogenes and tumor suppressor genes without global chromosome transformations, which only reflect the ongoing instability of the genome [29]. The described activation of SINEs and LINEs during the development of GB also contributes to carcinogenesis by mechanisms of influence on gene expression in cis and in trans, as well as by influencing specific microRNAs [33]. In particular, HERV-K sequences have been shown to inactivate the DNA repair *XRCC1* and *BRCA2* genes in GB [34]. Altered regulation of specific genes under the influence of certain HERVs can have a global impact on glioma carcinogenesis. For example, HERVs activate the *OCT4* gene, which correlates with the expression of key genes associated with glioma. High HERV levels were associated with a worse prognosis for patients and a higher grade of GB malignancy. Increased expression of *OCT4* and *SOX2* with a positive correlation was observed in GB stem cells. Thus, HERVs are important targets for controlling *OCT4* expression in GB carcinogenesis [35]. Activated HERVs during GB development can directly express oncogenic HERV proteins [21]. The HERV-W envelope protein has been shown to be produced by GB microglia cells in the tumor microenvironment. This protein modulates the behavior of GB cell lines in coculture with microglial cells, altering the expression of genes encoding cytokines and those involved in the regulation of GB cell proliferation and migration [36]. Thus, there is evidence of various pathways through which TEs stimulate carcinogenesis. Consequently, inhibition of TEs may be a promising strategy for the antitumor therapy of GB.

However, a converse phenomenon has been described, whereby activated TEs in GB tissues do not induce carcinogenesis, but rather promote the immune response against the tumor. These contradictions may be associated not with the methods used to study GB, but with the multidirectional mechanism of action of specific TEs distributed throughout the genome and therefore behaving differently. Limitations of research related to the use of various models (animal models and cell lines) must be taken into account. Therefore, to eliminate potential bias, it is necessary to compare the results of experimental studies with clinical data. This paradox can be explained by the involvement of different TEs in these processes: certain REs, when activated, promote GB carcinogenesis, while others, conversely, exert an anti-oncogenic effect. Identifying specific TEs with different effects on GB using bioinformatics methods is of interest for the development of GB treatments. Indeed, a comparative analysis of TE and satellite repeat expression was conducted between low-grade glioma and GB, revealing higher levels of LTR5 and HERV_a-int, as well as immune response-related genes. A total of 16 TEs are associated with slower progression of low-grade glioma (preventing tumor progression, respectively: LTR1/1A2/1C/1F2/7C/18C/27/27C/35A/40c/57/2752, and MER1B/9B/107/130), as well as 23 TEs are involved in shorter patient survival (promoting carcinogenesis progression, respectively: CR1-11_Crp, hAT-16_Crp, HERV1_LTRb, HERVI-int, HERVK3-int, L1M2a/1M3f, HSAT5, Kanga1c, L1-M4a1/M8/MB2/MDa, LTR18C/70/75, MER51-int/70-int/95/VKC4-int, MLT1E3-int, MSTC-int, and UCON97) [37]. Accordingly, the 16 specific LTR-containing REs described in this article exhibit antitumor activity, which is most likely associated with stimulation of the antitumor immune response by their expression products.

Single-cell transcriptome analysis using RNA sequencing from GB tissue samples revealed 370 peptides encoded by transposons and binding to human leukocyte antigens (HLA-I). The transcripts of LINE1, SINE-VNTR-Alus (SVA), and LTR REs were found to be the translation sources of these peptides. The expression of these peptides is specific for GB. Their use in tumor treatment is proposed [38], since anti-programmed cell death 1 (anti-PD-1) immunotherapy, which has been successful in other MNs, has proven ineffective in glioblastoma, despite enhancing local and systemic antitumor immunity [39]. GB patients develop resistance to TMZ, which is associated with low transcription of MER39B, LTR27B, and LTR81B. This is due to a positive correlation between the expression of these TEs and enhanced immune cell responses. Therefore, MER39B, LTR27B, and LTR81B can be used as GB biomarkers, whose levels determination predicts improved survival in patients receiving immunotherapy [13].

## Potential for glioblastoma therapy by targeting transposable elements

There are two diametrically opposed approaches to use TEs as targets in the treatment of GB: (1) inhibition of TEs that promote GB carcinogenesis and (2) activation of TEs whose expression products stimulate antitumor immunity. The first approach is based on the aforementioned data on the involvement of TEs in GB carcinogenesis, and therefore, TE silencing can suppress GB development. For this purpose, reverse transcriptase inhibitors are used, which exert an antitumor effect on GB cell lines [40, 41]. Because HML-2 is essential for maintaining GB stemness and for tumorigenesis in GB, it has been proposed as a unique therapeutic target for GB, as inhibition of HML-2 by antiretroviral drugs reduced reverse transcriptase activity, suppressing GB pluripotency and viability [23]. However, since the expression products of some TEs can stimulate antitumor immunity, inhibiting all REs may weaken the body’s fight against GB. Therefore, the most promising approach is to suppress specific TEs involved in GB carcinogenesis by targeting their promoter regions. Specific ncRNAs can be used as guides for such interventions.

Since many of the above described TEs contribute to GB carcinogenesis [15–17, 19–25], it can be suggested that nonspecific activation of TEs may contribute to the progression of GB. However, the most popular approach is epigenetic activation of TEs, which has demonstrated results in clinical and experimental studies. Because epigenetic abnormalities are reversible, the genes and proteins that control these events are promising targets for targeted tumor therapy. The possibility of treating GB by derepressing TEs is due not only to the activation of the immune response to TE expression products [38, 42], but also to the fact that not all TEs are involved in carcinogenesis. DNA methyltransferase inhibitors (DNMT-I) and histone deacetylase inhibitors (HDAC-I) have been proposed for the treatment of GB. By suppressing methylation, DNMT-I inhibitors, such as azatidine (5-aza) and decitabine, can activate the expression of not only TEs but also tumor suppressor genes [32].

HDAC inhibitors suppress oncogene transcription during the development of GB, thereby halting the cell cycle and tumor angiogenesis. According to a systematic review, HDAC inhibitors (vorinostat, and panobinostat) have shown some efficacy in phase I and II clinical trials for the treatment of GB [32]. This effect is mainly due to the activation of antitumor immunity by TEs expression products, and the method is called viral tumor mimicry [43]. Indeed, in PTEN-deficient GB, characterized by an immunosuppressive microenvironment and chemoresistance, coadministration of azacitidine with the epigenetic regulator EZH2 inhibitor (EZH2i) restored interferon signaling by reducing the levels of methylated histone H3K27me3 with subsequent transcriptional activation of ERVs [44]. Systemic administration of decitabine reactivated ERVs by stimulating interferon and major histocompatibility complex class I (MHC-I) responses, promoting enhanced immunotherapy in GB [45]. However, this approach has not found widespread application, which may be due to the nonspecific activation of expression of various TEs, including those that promote GB carcinogenesis. Therefore, a more targeted approach is needed, stimulating strictly defined immunogenic TEs not involved in GB carcinogenesis. This requires bioinformatics methods, the capabilities of which are currently limited. This approach requires the analysis of hundreds of thousands of different TEs distributed throughout the human genome, which comprise 46% of all its sequences.

The use of epigenetic modulators in GB therapy not only derepresses TEs but also enables their translation into unique peptides with immunogenic properties. The ability of TEs activated by epigenetic therapy to expand the antigen repertoire in GB was analyzed. Primary patient-derived GB stem cell lines were treated with DNMT and HDAC inhibitors, and TE transcripts induced by this method were determined. As a result, the ability of TEs to express antigens (unique peptides) that promote the development of an immune response was discovered [42]. A triple combination of TE derepression using epigenetic inhibitors and B-cell lymphoma-extra large (BCL-XL, an anti-apoptotic protein) with a monoclonal antibody against PD-1 was proposed, which suppressed GB growth in vivo with increased survival of GB mice [46].

An additional method for enhancing the antitumor immune response of GB may be the use of oncolytic viruses, whose action may also be mediated by TE activation. According to a systematic review, oncolytic therapy utilizes the herpesvirus herpes simplex virus 1 (HSV-1), adenovirus, reovirus, parvovirus H-1, and recombinant poliovirus [47]. It is also proposed to use the Zika virus in the treatment of GB [48]. Viruses used for oncolytic therapy are characterized by the ability to activate TEs of the human genome, which indicates the possible involvement of TEs in the immune mechanisms of this method. Thus, activation of HERVs has been identified under the influence of HSV-1 [49] and adenoviruses [50]. Indirect epigenetic induction of ERVs has been identified during combined reovirus treatment in the context of oncolytic therapy research [51]. Increased expression of ERVs has been identified in the development of vaccines against parvovirus infections [52].

## Relationship between transposable elements and microRNAs in glioblastoma development

For the treatment of glioblastoma, it is possible to use DNMT and HDAC inhibitors for epigenetic modulation and suppression of tumors [32]. Therefore, despite the effectiveness and stimulation of antitumor immunity, such a nonspecific approach may enhance carcinogenesis by activating TEs involved in this process and described above in this article (Figure 1). Since different TEs can influence the development of GB as oncogenes or tumor suppressors [37], a differentiated approach to targeted therapy of GB by activating TEs not involved in tumor pathogenesis and even those with an oncosuppressor role, with epigenetic silencing of TEs involved in development, is promising. For such purposes, the most suitable tools are microRNAs, which are able to control the expression of TEs not only post-transcriptionally but also transcriptionally, functioning as guides for DNMTs. This mechanism (RNA-directed DNA methylation), previously discovered in plants, was later discovered in humans in relation to certain REs [53] (Figure 2). The most suitable are microRNAs that are strictly complementary to specific TEs due to their common evolutionary origin. In order to search for such microRNAs, I analyzed the MDTE database on the origin of microRNAs from TEs [11] with data from the scientific literature on the possible involvement of such microRNAs in GB development. As a result, 43 TEs-derived microRNAs were identified whose expression was reduced (and therefore they had tumor suppressor properties) in GB according to clinical and experimental studies. The data obtained may further form the basis for the development of bioinformatics-based targeted therapy for GB by targeting TEs involved in carcinogenesis with simultaneous nonspecific epigenetic activation of TEs using a method called viral tumor mimicry.

**Figure 1:**
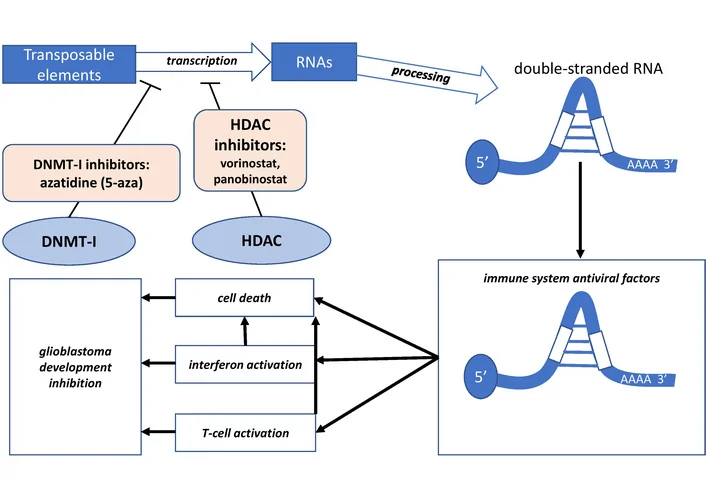
**Viral mimicry strategies in the treatment of glioblastoma (GB) with a differentiated approach in stimulation and inhibition of transposable elements (TEs).** HDAC: histone deacetylase; DNMT: DNA methyltransferase.

**Figure 2:**
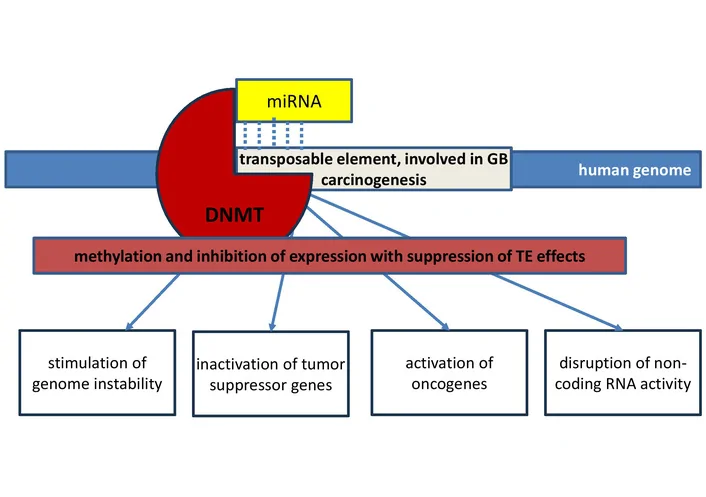
**Prospects for using microRNAs as guides for epigenetic silencing of transposable elements involved in glioblastoma carcinogenesis.** DNMT: DNA methyltransferase; GB: glioblastoma; TEs: transposable elements.

The potential of using tumor suppressor microRNAs is also linked to their pleiotropic antitumor activity, as a single microRNA can suppress the transcription of multiple target messenger RNAs (mRNAs, miRDB: https://mirdb.org), many of which contribute to the development of GB (Table 1). Back in 2019, microRNAs derived from TEs were identified using immunoprecipitation of GB tissues taken from patients. Many of these microRNAs arose from ancient LINE2s, which integrated into the human genome approximately 300 million years ago. These LINE2 microRNAs are formed from the 3’ end of the LINE2 consensus sequence and can therefore target transcripts containing LINE2 sequences in their 3’ untranslated region (3’-UTR). These transcripts can be generated not only from REs but also from protein-coding genes, regulating their expression. Compared with normal brain tissue, decreased levels of miR-95, miR-708-3p, miR-708-5p, and miR-1249-3p, as well as increased levels of miR-28, and miR-151a were detected [54]. The divergent changes in the levels of specific microRNAs in GB are associated with their ability to bind to transcripts of various genes involved in carcinogenesis. If the microRNAs target oncogene RNAs, these microRNAs exhibit tumor suppressor properties. They are characterized by low expression in the GB due to the adaptive nature of epigenetic changes during carcinogenesis when factors that inhibit tumor growth are suppressed. If the microRNA targets are tumor suppressor genes, these microRNAs are oncogenic and their expression in the GB is increased, as they promote tumor progression. Accordingly, examining the microRNAs derived from TEs, whose levels are reduced in GB, allows to consider tumor-suppressor microRNAs which potentially inhibit not only oncogenes but also the complementary transcripts of the TEs from which they originated.

**Table 1:** Transposable element-derived microRNAs whose expression is reduced in GB development.

**No.**	**MicroRNA, change in expression**	**TE-source [11]**	**Where microRNA was determined**	**Mechanism of influence on GB/target genes (miRDB)**
1	miR-95, ↓ [54–56]	LINE2	GB samples [56], GB cell lines [55]	Suppression of epithelial–mesenchymal transition [55]
2	miR-151a-3p (miR-151a), ↓ [57]	LINE2	GB cell lines [57]	Increases the sensitivity of cells to TMZ [57]
3	miR-224-3p, ↓ [58]	DNA/MER135	GB cell lines [58]	Inhibits *ATG5* expression, increases cell sensitivity to TMZ [58]
4	miR-224-5p, ↓ [59]	DNA/MER135	GB samples [59]	*CXCR4* inhibition [59]
5	miR-342-3p, ↓ [60]	SINE/tRNA-RTE	GB cell lines [60]	*Zic4* inhibition [60]
6	miR-345-5p, ↓ [61]	SINE/MIR	Blood plasma exosomes [61]	*EPN3, CTTNBP2NL, YEATS2, RFC1, RAB27B, ZNF133, RPA1, KMT5B*
7	miR-361-3p, ↓ [62]	DNA/hAT-Charlie	GB samples, GB cell lines [62]	*BCL2* inhibition [62]
8	miR-361-5p, ↓ [63]	DNA/hAT-Charlie	GB cell lines [63]	*ACTG1* inhibition [63]
9	miR-374a, ↓ [64]	LINE1	GB cell lines [64]	Stimulation of GB cells apoptosis [64]
10	miR-378a-3p, ↓ [65]	SINE/MIR	GB cell lines [65]	*TSPAN17* inhibition [65]
11	miR-422a, ↓ [66]	SINE/MIR	GB samples [66]	*PIK3CA* inhibition [66]
12	miR-513, ↓ [67]	DNA/hAT-Tip100	GB samples [67]	*IGF2BP1* inhibition [67]
13	miR-548x-3p (miR-548x), ↓ [68]	DNA/TcMar-Mariner	GB cell lines [68]	mTOR, RHEB, PDK1, PI3K/AKT inhibition [68]
14	miR-562, ↓ [69]	LINE1	GB samples [69]	c-MET/AKT signaling pathway inhibition [69]
15	miR-576-3p, ↓ [70]	LINE1	GB cell lines [70]	HIF-1α inhibition [70]
16	miR-579, ↓ [71]	DNA/TcMar-Mariner	GB cell lines [71]	*PDK1, Rheb, AKT1* inhibition [71]
17	miR-584-5p, ↓ [72]	DNA/hAT-Blackjack	GB samples, GB cell lines [72]	*KPNB1* inhibition [72]
18	miR-587, ↓ [73]	DNA/hAT-Tip100	GB samples [73]	*PDIA3* inhibition [73]
19	miR-588, ↓ [74]	LINE1	GB cell lines [74]	*ROBO1* inhibition [74]
20	miR-612, ↓ [75]	SINE/MIR	GB samples [75]	*AKT2* inhibition [75]
21	miR-619, ↓ [76]	SINE/Alu	GB samples [76]	*MDGA2, WBP1L, MAPK9, OGT, PTPRA, ACVR2B*
22	miR-625-5p (miR-625), ↓ [77]	LINE1	GB samples [77]	*AKT2* inhibition [77]
23	miR-634, ↓ [78]	LINE1	GB cell lines [78]	*CYR61* inhibition [78]
24	miR-646, ↓ [79]	LTR/ERVL	GB samples [79]	р62/Keap1/Nrf2 signaling and HO-1 inhibition [79]
25	miR-652-5p, ↓ [80]	DNA/hAT-Tip100	GB samples, GB cell lines [80]	SDC1/TGFβ2/pERBB4 signaling inhibition [80]
26	miR-708-3p, ↓ [54]	LINE2	GB samples [54]	*SCAMP1, JARID2, TRAF3, VIM, BAZ1B, SPRED1, HSPA4L*
27	miR-708-5p (miR-708), ↓ [81, 54]	LINE2	GB cell lines [81], GB samples [54]	*Akt1, Bcl2, CCND1, EZH2, MMP2, PARP-1* genes inhibition [81]
28	miR-885-3p, ↓ [82]	SINE/MIR	GB samples, GB cell lines [82]	*RGS8, GPCPD1, RAVER2, SOCS6, SPATS2L, RARG, ARMH3, NAT8L, IGF1R*
29	miR-1249-3p, ↓ [54]	LINE2	GB samples [54]	*GRB14, RGN, TBPL2, SPATA17, CCDC141, ADGRG2*
30	miR-1268a, ↓ [83]	SINE/Alu	GB samples [83]	*ABCC1* inhibition, increased sensitivity of GB to TMZ [83]
31	miR-1268b, ↓ [84]	SINE/Alu	GB samples [84]	*STXBP1, EEPD1, EGR4, CMIP, FCAR, HPD, USB1*
32	miR-1271, ↓ [85]	LINE2	GB samples [85]	CaMKII inhibition [85]
33	miR-1294, ↓ [86]	SINE/MIR	GB samples [86]	Inhibition of MYC and Wnt/β-catenin signaling [86]
34	miR-1303, ↓ [87]	SINE/Alu	GB samples [87]	*HOXA10* gene inhibition [87]
35	miR-1321, ↓ [88]	SINE/MIR	GB cell lines [88]	SERPINA1 inhibition [88]
36	miR-1825, ↓ [89]	LINE2	GB cell lines [89]	Wnt/β-catenin inhibition [89]
37	miR-3174, ↓ [90]	DNA/hAT-Charlie	GB samples, GB cell lines [90]	CDK6, PLAU, RHOA, MDM2, CD44 inhibition [90]
38	miR-3201, ↓ [91]	LINE2	GB samples [91]	*TENM1, SLITRK1, GCH1, NT5E, TMEM265, EPM2A*
39	miR-3664 (miR-3664-5p), ↓ [84]	DNA/TcMar-Tigger	GB samples [84]	*RIPOR1, ITK, MTDH, CAMTA2, TJP3, CCSER1, PTPRJ, LIX1*
40	miR-3681, ↓ [92]	LTR/ERVL	GB samples [92]	*MALT1* inhibition [92]
41	miR-4286, ↓ [93]	LTR/ERVL-MaLR	GB samples [93]	Regulates relationship between TGF-β and IGF-1 [93]
42	miR-4496, ↓ [94]	SINE/MIR	GB cell lines [94]	Suppresses epithelial–mesenchymal transition [94]
43	miR-7973, ↓ [95]	SINE/MIR	GB cell lines [95]	*ZC3H12B, NATD1, ZFYVE26, PXDN, IRAK4, RASL10A, GLE1*

↓: decrease in expression; TE: transposable element; GB: glioblastoma; TMZ: temozolomide; mTOR: mammalian target of rapamycin; HIF-1α: hypoxia-inducible factor-1α; CaMKII: calmodulin-dependent protein kinase II; TGF: transforming growth factor; SINE: short interspersed nuclear element; tRNA: transfer RNA; c-MET: cellular-mesenchymal-epithelial transition; HO-1: heme oxygenase-1; ERVL: endogenous retrovirus L; MaLR: mammalian apparent LTR-retrotransposon; RTE: retrotransposon encoded; MIR: mammalian-wide interspersed repeat; TSPAN17: tetraspanin 17.

LINE2 gave rise to miR-95 [11], which suppresses epithelial–mesenchymal transition and is characterized by low expression in the GB cell line [55]. Significant reductions in miR-95 levels were detected in stem cells from GB samples and correlated with poor patient survival [56]. LINE2-derived miR-151a-3p (miR-151a) [11] is expressed at higher levels in TMZ-sensitive GB cell lines than in TMZ-resistant cells. Transfer of miR-151a into GB cell lines via exosomes increased their sensitivity to the drug and was proposed as a treatment option for patients. Accordingly, miR-151a exhibits tumor suppressor properties [57]. MiR-224-3p (source is DNA-TE MER135 [11]) and its mimetics increase the sensitivity of GB cells to TMZ chemotherapy under hypoxic conditions. This microRNA also inhibits the expression of the *ATG5* oncogene, suppressing tumor cell mobility [58].

In plasma exosomes of patients with GB, a decrease in miR-345-5p levels was detected, while in patients with primary brain lymphoma, the levels of this microRNA were increased [61]. This indicates the multidirectional action of microRNAs depending on the type of MN, which is associated with the specific mechanisms of carcinogenesis of each tumor. The source of miR-345-5p is SINE/MIR [11]. LINE2 gave rise to miR-374a [11], the expression of which in GB cells stimulates their apoptosis [64]. MiR-378a-3p is derived from SINE/MIR [11]. MiR-378a-3p inhibits proliferation and migration of GB cells by silencing tetraspanin 17 (TSPAN17) [65]. Increased levels of miR-548o-3p were detected in the blood of patients with spinal cord GB [84]. MiR-548o-3p is originated from TcMar-Mariner DNA-TE [11].

Derived from the TcMar-Mariner DNA transposon, miR-548x-3p (miR-548x) [11] suppresses GB cell proliferation by inhibiting PI3K/AKT signaling. This microRNA targets mRNAs of *mTOR, RHEB*, and *PDK1* oncogenes [71]. The source of miR-579 is the TcMar-Mariner DNA-TE [11]. MiR-579 is expressed at low levels in GB cell lines. MiR-579 targets *PDK1, RHEB*, and *AKT1* oncogenes [71]. LINE1-derived miR-562 [11] is expressed at low levels in GB samples from patients. MiR-562 targets the mRNA of the c-MET oncogene, negatively regulating the c-MET/AKT signaling pathway, thereby regulating cell proliferation. MiR-562 also inhibits caspase-3 expression [69].

LINE1s are also sources of miR-576-3p [11], which inhibits the migration and proangiogenic capacity of GB cells activated by hypoxia and hypoxia-inducible factor-1α (HIF-1α) because it interacts with the 3’-UTR of *HIF-1α* gene [70]. LINE1-derived miR-588 [11] regulates GB cell invasion and migration (in cell lines) by inhibiting *ROBO1* oncogene expression [74]. A study of intratumor microRNA heterogeneity suggests a role for Alu-derived miR-619 [11] in the development of GB. Levels of miR-619 are reduced in GB tissues, suggesting that it exhibits tumor suppressor properties [76]. LINE1-derived miR-625-5p [11] suppresses GB cell proliferation and enhances chemotherapy sensitivity by targeting AKT2 in GB tissues and GB cell lines [77].

LINE1 is also a source of miR-634 [11], the expression of which was reduced in LN229 glioblastoma cells. The target of the tumor suppressor miR-634 is the CYR61 matricellular protein, which regulates the proliferation and migration of GB cells [78]. LTR/ERVL generates miR-646 [11], which inhibits GB cell proliferation and migration by interacting with sequestosome 1 (p62) in the 3’-UTR, attenuating *HO-1* gene expression and regulating the p62/Keap1/Nrf2 signaling pathway. In GB tissues, miR-646 levels are lower than in normal tissues [79]. MiR-652-5p, derived from the hAT-Tip100 DNA-TE, is implicated in the pathogenesis of GB [11]. Hypermethylation of the miR-652-5p gene promoter enhances GB malignancy under hypoxic conditions in experimental and GB tissue samples by affecting the SDC1/transforming growth factor β 2 (TGFβ2)/pERBB4 signaling pathways [80]. LINE2-derived miR-708-5p [11] functions as a tumor suppressor in GB cell culture. Increased miR-708 levels inhibit proliferation and invasion of GB cells by inducing apoptosis and also reduce the expression of Akt1, Bcl2, CCND1, EZH2, MMP2, and PARP-1 proteins involved in GB pathogenesis [81].

Alu is the source of miR-1268a [11], which regulates the expression of ABCC1/MRP1 (a membrane transporter that promotes chemoresistance) and thus contributes to increased sensitivity of GB to TMZ. Levels of this tumor-suppressor microRNA are reduced in GB tissues [83]. LINE2-derived miR-1271 [11] suppresses GB cell proliferation and invasion by regulating calcium/calmodulin-dependent protein kinase II (CaMKII) expression in GB tissues from patients with decreased miR-1271 levels. Accordingly, CaMKII oncogene levels are increased in GB [85]. Stimulation of miR-1271 expression has been proposed to increase the sensitivity of GB to chemotherapy, since its target is *Bcl-2* mRNA, which promotes chemoresistance [96].

SINE/MIR-derived miR-1321 [11] exhibits anti-oncogenic properties in experiments on GB cells by inhibiting the expression of the *SERPINA1* oncogene [88]. LINE2 is the source of miR-1825 [11], the levels of which are reduced in GB tissues. Stimulation of miR-1825 expression in GB cell lines suppresses GB progression by silencing the *CDK14* oncogene and inhibiting the Wnt/β-catenin signaling pathway. Accordingly, this microRNA regulates proliferation, migration, and apoptosis of GB cells [89]. In vivo and in vitro studies have shown a decrease in miR-3174 expression in GB tissues and GB cell lines [90]. The targets of this microRNA in the mechanisms of GB carcinogenesis are mRNAs of *CDK6, PLAU, RHOA, MDM2,* and *CD44* genes [90]. MiR-3174 is originated from hAT-Charlie DNA-TE [11]. MiR-3201 originated from LINE2 [11]. MiR-3201 is expressed at low levels in GB tissues and is downregulated by direct current electric field in GB cell culture experiments [91]. MiR-3664 [11], derived from the TcMar-Tigger DNA transposon, is implicated in the progression of spinal cord carcinogenesis in patients with spinal cord GB. MiR-3664 and miR-1268b are derived from Alu [11]. Levels of these microRNAs are reduced in GB tissues [84].

LTR/ERVL-MaLR-derived miR-4286 [11] is involved in the connections between TGF-β and IGF-1 signaling pathways during epithelial–mesenchymal transition and GB invasion. Elevated levels of miR-4286 significantly attenuate the expression of IGF-1-mediated mesenchymal markers, TGFβ1, and TGFβR2. A study of GB patients demonstrated the tumor suppressor function of miR-4286 and reduced levels of it in tumors [93]. SINE/MIR is the source of miR-4496 [11], mimetics of which exert antitumor effects on GB cell lines. At the same time, the cytoskeleton-associated protein 2-like (CKAP2L) protein inhibits the expression of miR-4496, exerting an oncogenic effect, stimulating epithelial–mesenchymal transition, proliferation, migration and invasion of tumor cells [94]. SINE/MIR-derived miR-7973 [11] is characterized by low expression in the GB cell line and, accordingly, exhibits tumor suppressor properties [95]. Figure 3 shows a diagram of the microRNAs involved in GB development grouped by functional groups, showing the origin of these microRNAs from specific TEs.

**Figure 3:**
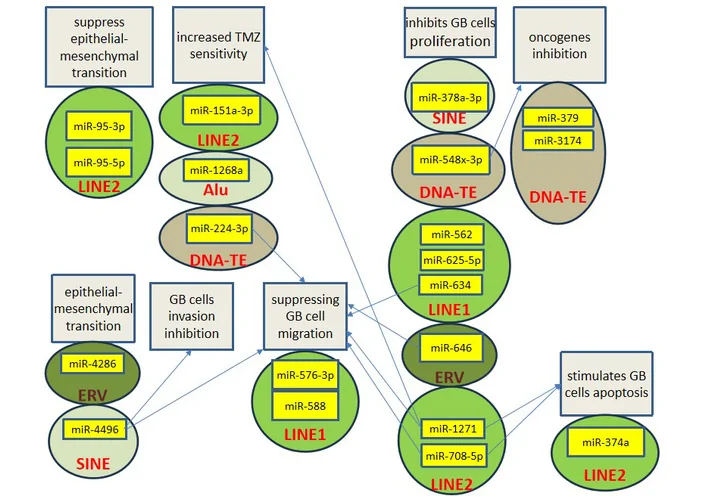
**Scheme of functional groups of oncosuppressive microRNAs associated with glioblastoma.** GB: glioblastoma; TMZ: temozolomide; SINE: short interspersed nuclear element; TE: transposable element; ERV: endogenous retrovirus.

Of interest is also the examination of microRNAs derived from TEs and exhibiting oncogenic properties in GB (Table 2). For targeted therapy of GB, it is possible to use such microRNAs as targets for antisense oligonucleotides (ASOs), which are already used in oncology to treat various MNs [97]. Increased expression of such microRNAs may be due to their formation directly from TE transcripts through maturation by a system of enzymes [11]. However, additional experimental and clinical studies are needed before these molecules can be considered as therapeutic targets.

**Table 2:** TE-derived microRNAs, whose expression is increased in glioblastoma development.

**No.**	**MicroRNA, expression changes**	**TE source [11]**	**Where microRNA was determined**	**Mechanism of influence on GB/target genes (miRDB)**
1	miR-28-5p, ↑ [54, 98]	LINE2	GB samples, GB cell lines [54, 98]	Inhibition of *FOXO1* tumor suppressor [98]
2	miR-151a, ↑ [54]	LINE2	GB samples [54]	*AGO2, RPS6KA5, ME1, YTHDF3, PITPNA, CASD1, CLASP2*
3	miR-320d, ↑ [99]	LINE1	GB samples [99]	*YOD1, CDKL5, ONECUT2, SPOPL, SH2B3, KITLG, GPBP1*
4	hsa-miR-330-5p, ↑ [76]	SINE/MIR	GB samples [76]	*CEP85, GPD2, LETM2, PALM, YBY, ETS1, UBXN10, KCNIP2*
5	miR-340-5p, ↑ [100]	DNA/TcMar-Mariner	Blood [100]	*GTF2E2* [101]
6	miR-374b-3p, ↑ [102]	LINE2	GB cell lines [102]	Stimulation of M2 macrophage polarization [102]
7	miR-374b-5p, ↑ [103]	LINE2	GB cell lines [103]	*GATA3, SEMA3B* silencing [103]
8	miR-548b, ↑ [104]	DNA/TcMar-Mariner	GB samples [104]	FoxM1 inhibition [104]
9	miR-548o-3p, ↑ [84]	DNA/TcMar-Mariner	Blood [84]	*TRAPPC8, CASP8AP2, GPM6A, STRN, GLI3, ZMAT1, ZBTB44, CNTNAP4*
10	miR-571, ↑ [84]	LINE1	Blood [84]	*REG1A, LAMA1, ZNF426, KCNH7, SCRT2, SPAG8*
11	miR-582-5p, ↑ [105]	LINE/CR1	GB samples, GB cell lines [105]	Prevents apoptosis; Bim, caspase 3 and caspase 9 inhibition [105]
12	miR-603, ↑ [106]	DNA/TcMar-Mariner	GB samples, GB cell lines [106]	*CTNNBIP1* and *WIF1* inhibition [106]
13	miR-626, ↑ [107]	LINE1	Blood serum [107]	*PAPOLB, DPY19L3, CDH13, BCL11A, SLAMF7, SMCP*
14	miR-640, ↑ [108]	SINE/MIR	GB samples, GB cell lines [108]	*SLIT1* inhibition [108]
15	miR-1246, ↑ [109]	LTR/ERVL-MaLR	Plasma and GB exosomes [109]	Stimulation of differentiation myeloid suppressor cells [109]
16	miR-1290, ↑ [110]	DNA/TcMar-Tigger	GB samples [110]	*SGO1, RTKN2, HTRA3, OSBPL6, EHHADH, PALM2-AKAP2, AKAP2, ACER3*
17	miR-4431, ↑ [84]	ERVL-MaLR	Blood [84]	*MITD1, PRAMEF26, SIX3, RNF111, CATSPERZ*
18	miR-4448, ↑ [111]	LTR/ERVK	GB cell lines [111]	*PAN3, FAM177A1, ARID2, BPTF, CHD2, SOX7, ZNFX1*
19	miR-4703-3p, ↑ [84]	DNA/TcMar-Tigger	Blood [84]	*CLDN20, EXTL2, CDYL, ZNF239, PHF12, ZNF470, SATL1, DYNAP, MITF*
20	miR-6088, ↑ [112]	SINE/Alu	GB samples, GB cell lines [112]	*SMARCB1* inhibition [112]

↑: increased expression; TE: transposable element; GB: glioblastoma; SINE: short interspersed nuclear element; ERVL: endogenous retrovirus L; MaLR: mammalian apparent LTR-retrotransposon.

LINE2 is the source of miR-28-5p [11], whose expression is elevated in GB patient tissues and GB cell lines. This microRNA promotes tumor spheroid formation, stimulating GB cell survival and proliferation. MiR-28-5p targets the mRNA of the *FOXO1* tumor suppressor gene [98]. LINE1-derived miR-320d [11] is highly expressed in GB tissues [99]. Increased levels of miR-330-5p (source is SINE/MIR [11]) were detected in GB samples [76]. MiR-374b-3p is derived from LINE2 [11]. This microRNA promotes angiogenesis and GB progression by stimulating M2 macrophage polarization [102]. In a study of GB samples and GB cell lines, elevated levels of miR-374b-3p were detected in exosomes derived from GB stem cells [102]. MiR-374b-5p inhibits GATA3 mRNA, thereby suppressing SEMA3B tumor suppressor [103].

In blood samples from patients with spinal cord GB, an increased concentration of miR-571 was detected [84]. MiR-571 originated from LINE1 [11]. LINE/CR1 gave rise to miR-582-5p [11], which inhibits apoptosis by promoting GB stem cell survival through inhibition of Bim, caspase 3, and caspase 9. This oncogenic microRNA is expressed at high levels in GB tissues [105]. MiR-603, derived from the TcMar-Mariner DNA-TE [11], is overexpressed in GB tissues and cell lines. MiR-603 enhances nuclear β-catenin production by activating the Wnt/β-catenin signaling pathway. Accordingly, this microRNA stimulates the proliferation and survival of GB cells. MiR-603 targets CTNNBIP1 and WIF1 [106]. Levels of LINE1-derived miR-626 [11] in the serum of GB patients are significantly elevated [107].

Oncogenic miR-640 (source is SINE/MIR [11]) promotes GB cell proliferation and adhesion by inhibiting SLIT1 expression. In GB tissues and cell lines, miR-640 levels are elevated [108]. LTR/ERVL-MaLR-derived miR-1246 [11] in patient plasma exosomes stimulated the differentiation and activation of myeloid-derived suppressor cells in extracellular signal-regulated kinase (ERK) and dual-specificity phosphatase (DUSP3)-dependent ways. Under hypoxic conditions, miR-1246 levels in GB-derived exosomes increased [109]. TcMar-Tigger DNA-TE-derived miR-1290 [11] is characterized by increased expression in GB tissues taken from patients [110]. In blood samples from patients with spinal cord GB, an increased concentration of miR-4431 was found [84]. MiR-4431 originated from ERVL-MaLR [11]. LTR/ERVK gave rise to miR-4448 [11], which is expressed at elevated levels in GB cell culture [111]. In blood samples from patients with spinal cord GB, an increased concentration of miR-4703-3p was detected [84], which originated from the TcMar-Tigger DNA transposon [11]. Figure 4 shows a diagram of oncogene microRNAs involved in GB development grouped by functional groups, showing the origin of these microRNAs from specific TEs.

**Figure 4:**
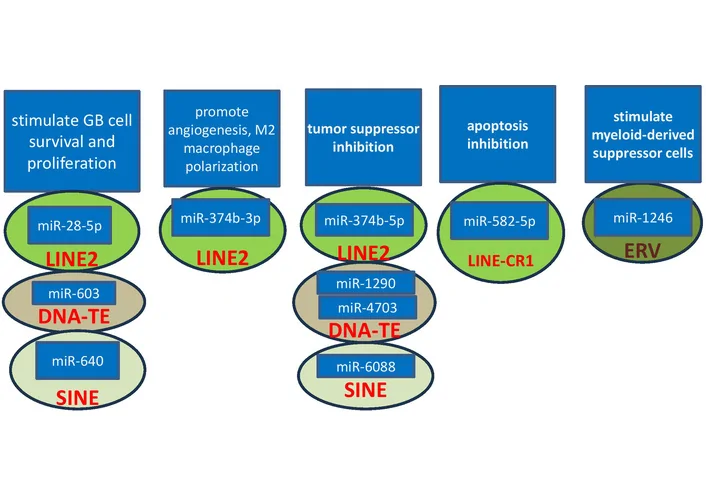
**Scheme of functional groups of oncogenic microRNAs associated with glioblastoma.** GB: glioblastoma; SINE: short interspersed nuclear element; TE: transposable element; ERV: endogenous retrovirus.

Despite the fact that DNA-TEs occupy less than 5% of the human genome, this class of TEs contains a large number of diverse families of different sizes, the most common of which are hAT-Charlie (1.59% of the genome), TcMar-Tigger (1.29% of the genome), hAT-Tip100 (0.41%), and TcMar-Mariner (0.098%) [6]. The data presented in Tables 1 and 2 on the role of DNA-TE-derived microRNAs (12 of 41 tumor suppressor (29%) and 6 of 19 oncogenic microRNAs (31.6%)) indicate a possible indirect influence of these TEs on the development of GB. Indeed, the Sleeping Beauty (SB) DNA-TE is widely used in experimental studies. Mobilization of SB from the Rosa26 locus in mice has been found to lead to gliomagenesis. By cloning insertions from additional GBs generated by SB mobilization in the Rosa26 compartment, candidate genes involved in GB development were identified. Changes in the expression of these genes are associated with SB insertions in their region. Comparison with the genetic and epigenetic features of mRNA expression in human GB revealed that these genes are oncogenes and tumor suppressors in the development of human GB: *ABTB2, ARID1B, CHD7, CREBBP, CSF1, DYRK1A, ELOVL6, FAF1, FLI1, GRAMD1C, HEPACAM2, KPNA4, LSAMP, MIR101C, MKLN1, NAV3, NFIA, PAG1, PPP3R1, RBPJ, RPS6KA5, SMAD4, TANC2, TSG101, USP9X, ZFP212* [113]. Furthermore, DNA-TEs can be used as tools for delivering specific genes into GB cells and integrating them into the genome. In experiments on immunodeficient mice with xenografted GB, SB-mediated transfer of antiangiogenic genes significantly increased the survival of animals with established tumors. However, delivery of these genes via episomal plasmids proved ineffective. SB-mediated gene therapy may improve immunogen therapy by sustaining cytokine expression in GB tissues. In particular, integration of the interferon (IFN-γ) gene using this method increased the expression of MHC-I antigens, enhanced the activity of T cells and natural killers, inhibiting angiogenesis and GB growth [114].

## Relationships of transposable element-derived microRNAs with other noncoding RNAs

In evolution, TEs have been the origin of diverse genes encoding proteins, long noncoding RNAs (lncRNAs), and circular RNAs (circRNAs) [115]. This is reflected in the fact that 82.5% of lncRNA transcripts contain at least one TEs sequence. LncRNAs are molecules larger than 200 nucleotides that, like protein-coding gene transcripts, are characterized by 5’ capping, 3’ polyadenylation, and splicing. Depending on the location of their genes, lncRNAs are classified as intergenic (lincRNA), intronic, antisense (transcribed from the complementary chain of protein-coding genes), and bidirectional (formed from bidirectional transcripts of protein-coding genes) [11]. CircRNAs are a type of ncRNAs that form a closed loop structure and are formed by covalently linking the 5’ and 3’ ends of the RNA. The presence of complementary sequences to microRNAs allows them to bind and inhibit their function [86, 87]. In this regard, circRNAs function as competing endogenous RNAs (ceRNAs), displacing microRNAs from their mRNA targets, thereby regulating gene expression. Due to the lack of free ends, circRNAs are extremely stable and resistant to degradation. They are tissue-specific and are expressed in highest quantities in the human brain. With respect to cancer, circRNAs can exhibit oncogenic or tumor-suppressive properties [66].

The tumor suppressor miR-224-5p, derived from the DNA-TE MER135 [11], is a target of the oncogenic circFGFR1 in GB development. This circRNA serves as a sponge for miR-224-5p, thereby increasing the expression of CXCR4, which is regulated by miR-224-5p [59]. MiR-340-5p (source is TcMar-Mariner DNA-TE [11]) is expressed at elevated levels in the blood of patients with GB [100]. The target of miR-340-5p is the mRNA of the *GTF2E2* gene, the protein of which inhibits the development of GB [101]. Experiments on GB cell lines revealed that miR-340-5p is a target of circ_0012381, which induces M2 polarization of microglial macrophages by increasing ARG1 levels. *ARG1* expression is directly inhibited by miR-340-5p [116]. Also, miR-340 targets in the development of GB are mRNAs of Sox2 and Bcl-w [117].

The hAT-Charlie DNA-TE-derived tumor suppressor miR-361-3p [11] interacts with the oncogenic circRNA SERPINE2 in GB tissues and cell lines. MiR-361-3p targets mRNA of the anti-apoptotic protein gene *BCL2* [62]. SINE/MIR is the source of miR-422a [11], which exhibits tumor suppressive properties during the development of GB. High concentrations of miR-422a are detected in normal brain, whereas expression of this microRNA is reduced in GB tissues. Accordingly, increased miR-422a production reduces the oncogenic properties of GB cells in vitro and in vivo. MiR-422a targets the mRNA of the PIK3CA gene, a key player in the PI3K/Akt signaling pathway, which is often dysregulated in GB. MiR-422a interacts with circNT5E, which is highly expressed in GB tissues and cell lines [66]. MiR-548b [11], which promotes GB development by inhibiting FoxM1 [104], originated from the TcMar-Mariner DNA-TE. This microRNA is a target of circBFAR [104].

Tumor suppressor miR-584-5p (source is hAT-Blackjack DNA-TE [11]) inhibits the expression of the *KPNB1* oncogene, suppressing cell proliferation, migration, invasion, and cell cycle in GB tissues and cell lines. The circRNA circPITX1 functions as a sponge for miR-584-5p [72]. The hAT-Tip100 DNA-TE-derived miR-587 [11] is a target of circRFX3, which acts as a ceRNA for this microRNA and stimulates GB progression. Since miR-587 targets PDIA3 mRNA, circRFX3 increases PDIA3 protein expression [73]. The genomic source of miR-1294 is SINE/MIR [11]. This microRNA is expressed at low levels in GB tissues and is inhibited by the oncogenic circRNA circ-AHCY. By acting as a sponge for miR-1294, circ-AHCY activates the Wnt/β-catenin signaling pathway, as miR-1294 targets the *MYC* oncogene [86]. MiR-1303 is characterized by interaction with circGRIK2, which stimulates GB [87]. MiR-1303 is originated from Alu [11]. MiR-1303 targets HOXA10 directly, whereas circGRIK2 blocks miR-1303, leading to increased HOXA10 expression [87]. Thus, 9 TEs-derived microRNAs have been described that are involved in GB development and interact with circRNAs in these mechanisms (Table 3).

**Table 3:** Relationships between TE-derived microRNAs, lncRNAs and circRNAs.

**No.**	**MicroRNA expression changes in GB**	**TE-source [11]**	**Where microRNA was determined**	**Noncoding RNA that interacts with microRNA**
1	miR-224-5p, ↓ [59]	DNA/MER135	GB sample [59]	Oncogenic circFGFR1 [59]
2	miR-340-5p, ↑ [100]	DNA/TcMar-Mariner	Blood [100]	Tumor suppressor circ_0012381 [115]
3	miR-342-3p, ↓ [60]	SINE/tRNA-RTE	GB cell lines [60]	Linc-00313 [118]
4	miR-361-3p, ↓ [62]	DNA/hAT-Charlie	GB sample, GB cell lines [62]	Oncogenic circRNA SERPINE2 [62]
5	miR-361-5p, ↓ [63]	DNA/hAT-Charlie	GB cell lines [63]	Oncogenic lncRNA COX10-AS1 [63]
6	miR-422a, ↓ [66]	SINE/MIR	GB sample [66]	Oncogenic circNT5E [66]
7	miR-513, ↓ [67]	DNA/hAT-Tip100	GB sample [67]	Oncogenic lncRNA PCAT6 [67]
8	miR-548b, ↑ [104]	DNA/TcMar-Mariner	GB sample [104]	Tumor suppressor circBFAR [104]
9	miR-584-5p, ↓ [72]	DNA/hAT-Blackjack	GB sample, GB cell lines [72]	Oncogenic circPITX1 [72]
10	miR-587, ↓ [73]	DNA/hAT-Tip100	GB sample [73]	Oncogenic circRFX3 [73]
11	miR-612, ↓ [75]	SINE/MIR	GB sample [75]	Oncogenic LINC00152 [75]
12	miR-885-3p, ↓ [82]	SINE/MIR	GB sample, GB cell lines [82]	Oncogenic lncRNA HOXB-AS1 [82]
13	miR-1294, ↓ [86]	SINE/MIR	GB sample [86]	Oncogenic circ-AHCY [86]
14	miR-1303, ↓ [87]	SINE/Alu	GB sample [87]	Oncogenic circGRIK2 [87]
15	miR-3681, ↓ [92]	LTR/ERVL	GB sample [92]	Oncogenic lncRNA HOXD-AS2 [92]
16	miR-6088, ↑ [112]	SINE/Alu	GB sample, GB cell lines [112]	Tumor suppressor lncRNA MEG3 [112]

↓: decreased expression; ↑: increased expression; GB: glioblastoma; TE: transposable element; SINE: short interspersed nuclear element; tRNA: transfer RNA; lncRNA: long noncoding RNA; circRNAs: circular RNAs; ERVL: endogenous retrovirus L; RTE: retrotransposon encoded.

An analysis of the scientific literature also revealed the relationship between 7 TEs-derived microRNAs and lncRNA molecules in the mechanisms of GB carcinogenesis. MiR-342-3p, derived from SINE-tRNA-RTE [11], is detected at reduced levels in the blood of GB patients [60]. Using GB cell lines, miR-342-3p was identified as a target for the lncRNA Linc-00313, which stimulates GB development and serves as a competitive endogenous RNA for this microRNA. The target of miR-342-3p is the mRNA of the *Zic4* gene. Reverse cross-regulation was also observed, as Zic4 binds to the UPF1 and Linc-00313 promoters, increasing their expression. This results in the formation of a chain: Linc-00313>miR-342-3p>Zic4>Linc-00313 [118]. Tumor suppressor miR-361-5p, derived from the hAT-Charlie DNA-TE [11], inhibits the expression of the *ACTG1* oncogene, which suppresses apoptosis and stimulates proliferation of GB cells, in experiments on GB cell lines. Antisense lncRNA COX10-AS1 interacts with miR-361-5p like a sponge, causing ACTG1 derepression [63]. LncRNA PCAT6 enhances GB progression by binding to the tumor suppressor miR-513, which targets the *IGF2BP1* (insulin-like growth factor 2 mRNA binding protein 1) oncogene [67]. The source of miR-513 is the DNA-TE hAT-Tip100 [11].

SINE/MIR-derived miR-612 [11] is a target of the oncogenic LINC00152, which promotes the development of GB by interfering with the AKT2/NF-κB signaling pathway. This mechanism is mediated by miR-612’s inhibition of AKT2 [75]. In GB tissues and cell lines, the lncRNA HOXB-AS1 is highly expressed, the inhibition of which causes cell cycle arrest, migration and proliferation of GB cells. HOXB-AS1 functions as a ceRNA for the tumor suppressor miR-885-3p, whose levels are reduced in GB [82]. MiR-885-3p is derived from SINE-MIR [11]. MiR-3681, derived from LTR/ERVL [11], is a target of lncRNA HOXD-AS2, which promotes proliferation, migration, and invasion of GB cells. The tumor suppressor miR-3681-5p targets *MALT1* oncogene mRNA [92]. Tumor suppressor lncRNA MEG3 in experiments on GB cell lines interacts with oncogenic miR-6088, thereby increasing SMARCB1 expression. SMARCB1 protein suppresses proliferation and migration of GB cells, and the mRNA of this protein is a target of miR-6088, while low expression of MEG3 is observed during GB culture growth [112]. The source of miR-6088 is Alu [11].

Most microRNAs interacting with lncRNAs and circRNAs are characterized by low levels in GB and tumor-suppressive mechanisms of action. This is also consistent with the observed similar trend in TE-derived microRNAs in GB development (43 microRNAs with low expression and 20 with high expression). It can be hypothesized that in carcinogenesis, lncRNA and circRNA molecules reflect an ancient property of the TE transcripts from which they evolved: to inhibit host defense mechanisms against their own proliferation. These host mechanisms include the RNA interference system which produces short ncRNAs. Thus, TE-derived microRNAs involved in the pathogenesis of GB may be targets of both lncRNAs and circRNAs, suggesting complex epigenetic mechanisms of GB development. Furthermore, TE transcripts themselves can also function as ceRNAs, interacting with their derived microRNAs and neutralizing their mechanisms of action. This is logical given the 43 TEs-derived microRNAs described in this article, whose expression is reduced in GB. This suggests a complex system of interactions between microRNAs, lncRNAs, circRNAs, and TEs transcripts (Figure 5). Targeting this system with specific molecules (microRNA mimetics or ASOs) is promising, and further bioinformatics research is needed to identify these.

**Figure 5:**
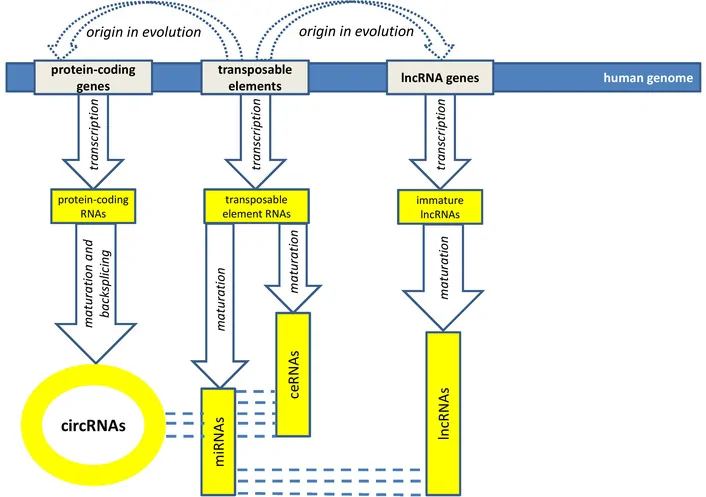
**MicroRNAs as objects for complementary interaction with several molecules.** circRNAs: circular RNAs; ceRNAs: competing endogenous RNAs; lncRNAs: long noncoding RNAs.

## Conclusion

There are still many unresolved issues in the study of GB, including a lack of precise data on how many and which REs (their location in the genome) contribute to carcinogenesis, and which, on the contrary, trigger an immune response against the tumor. Therefore, further bioinformatics studies are needed to identify the localization of specific TEs in the human genome that stimulate glioblastoma growth and progression. In this regard, it is promising to create a map of the distribution of all known TEs across the loci of the human genome, which would allow identifying changes in their expression during the development of GB. Currently, such resources are limited, as TEs occupy nearly half of the human genome and are distributed across intergenic, intronic, and regulatory regions. Bioinformatics research in this area could enable the use of specific microRNAs derived from TEs and strictly complementary to their sequences as guides for epigenetic inhibition by recruiting DNA methyltransferase. However, additional experimental and clinical studies are needed before these molecules can be considered as therapeutic targets. The challenge of viral mimicry in the treatment of GB is the lack of methods for targeted stimulation of only those TEs that are not involved in GB carcinogenesis, but whose transcripts and proteins possess immunogenic properties for antitumor action. Therefore, developing methods for activating specific TEs without stimulating carcinogenesis, while simultaneously inhibiting oncogenic TEs and ncRNAs, could help patients with GB. In the future, it is possible to integrate the work of bioinformaticians, specialists in TEs and microRNA, and epigenetics to develop effective approaches to specific targeted therapy for GB.

GB is one of the most aggressive malignancies with pharmacoresistance and poor prognosis for patients. The prospects for GB therapy may be related to the study of the role of TEs and their relationships with epigenetic factors, especially with microRNAs, lncRNAs and circRNAs. An analysis of the scientific literature indicates hyperactivation of various TEs (LINE1s, Alus, and ERVs) during GB development and the direct effect of activated TEs on GB progression. This effect is due to the formation of genomic instability due to recombinations between TEs in the genome, epigenetic changes due to exposure to TEs expression products, activation of oncogenes and inactivation of tumor suppressors due to TEs insertions into their loci. To eliminate these oncogenic factors, TEs inhibition is promising in GB therapy. However, increased expression of some ERVs in GB samples may stimulate antitumor immunity. Therefore, a differentiated approach is needed with specific inhibition of TEs involved in GB pathogenesis and simultaneous stimulation of immunoreactive ERVs. As tools for such effects, it is possible to use tumor suppressor microRNAs involved in the pathogenesis of GB and strictly complementary to certain TEs. An analysis of the scientific literature made it possible to describe 43 such microRNAs that originated from TEs and are tumor suppressors. Twenty oncogenic microRNAs derived from TEs and involved in GB development are also identified, which can be used as targets for targeted GB therapy. However, additional experimental and clinical studies are needed before these molecules can be considered as therapeutic targets. This is due to the complex relationships of the described microRNAs with other ncRNAs and epigenetic factors. The article describes interactions of 16 microRNAs derived from TEs and interacting with lncRNAs and circRNAs.

## Abbreviations

3’-UTR: 3’ untranslated region
ASOs: antisense oligonucleotides
CaMKII: calmodulin-dependent protein kinase II
ceRNAs: competing endogenous RNAs
circRNAs: circular RNAs
DNMT: DNA methyltransferase
ERVs: endogenous retroviruses
GB: glioblastoma
GWAS: genome-wide association studies
HDAC-I: histone deacetylase inhibitors
HERV: human endogenous retrovirus
HIF-1α: hypoxia-inducible factor-1α
HSV-1: herpes simplex virus 1
LINEs: long interspersed nuclear elements
lncRNAs: long noncoding RNAs
LTRs: long terminal repeats
MDTE: microRNAs derived from transposable elements
MN: malignant neoplasm
mRNAs: messenger RNAs
ncRNA: noncoding RNA
PD-1: programmed cell death 1
REs: retroelements
SINEs: short interspersed nuclear elements
TEs: transposable elements
TMZ: temozolomide
TSPAN17: tetraspanin 17

## Declarations

### Copyright

© The Author(s) 2026.
